# Recruitment and Data Management of the Chinese Hearing Solution for Improvement of Cognitive Function in Elders (CHOICE) Cohort: Baseline and Follow‐up Assessment of Hearing and Cognition

**DOI:** 10.1002/alz70857_096890

**Published:** 2025-12-24

**Authors:** Ying Chen, Zaichao Wu, Jie Chen, Feifan Zhao, Qiongfei Yu, Hao Wu

**Affiliations:** ^1^ Shanghai Ninth People's Hospital, Shanghai Jiaotong University School of Medicine, Shanghai, Shanghai, Shanghai, China; ^2^ Ear Institute, Shanghai Key Laboratory of Translation Medicine on Ear and Nose Disease, Shanghai Jiaotong University School of Medicine, Shanghai, Shanghai, Shanghai, China; ^3^ Department of Otolaryngology‐Head and Neck Surgery, Shanghai Ninth People's Hospital, Shanghai Jiaotong University School of Medicine, Shanghai, China., Shanghai, Shanghai, China; ^4^ Ear Institute, Shanghai Key Laboratory of Translation Medicine on Ear and Nose Disease, Shanghai Jiaotong University School of Medicine, Shanghai, China., Shanghai, Shanghai, China

## Abstract

**Background:**

Age‐related hearing impairment is a prevalent condition among the elderly and has been proven associated with cognitive decline. The Chinese Hearing Solution for Improvement of Cognitive Function in Elders ‐ Cohort (CHOICE‐C) is a multi‐center, prospective cohort designed to explore the link between hearing impairment and cognitive decline. This paper outlines the recruitment, quality control, and data management processes of CHOICE‐C study, presenting baseline and first‐year follow‐up results

**Method:**

Recruitment was conducted via routine physical exams at community health centers, along with community outreach and social media campaigns. Participants were instructed to complete a questionnaire covering demographic details, hearing history and symptoms, as well as Mini‐Mental State Examination (MMSE) for cognitive assessment. A battery of audiological assessments were performed, including acoustic immittance, pure‐tone audiometry, and Speech‐in‐Quiet Test (SIQT). And multi‐stage quality control measures were implemented to ensure the reliability and accuracy of the collected data

**Result:**

From May 2021 to September 2024, 18,982 elderly aged over 60 years old were recruited. After exclusion of individuals with missing questionnaire or audio‐metric data, 18,714 participants were retained (mean age: 69.6± 6.26 yrs; 56% female). 53.3% of baseline participants have completed the first follow‐up. Among the participants, 73% have hearing impairment with a better‐ear pure tone average (BPTA) above 20 dB HL. Compared to individuals with normal hearing, they were more likely to be older, male, less educated, to report tinnitus, and to exhibit lower MMSE scores. We found that alcohol consumption (OR=1.28, 95% CI 1.03‐1.58) and self‐reported hearing impairment (OR=1.21, 95% CI 1.06‐1.40) were associated with improved hearing and cognition when setting group as reference with constant/decreased hearing and constant cognitive level. Approximately 5–17.3% of questionnaire interviews and 5% of audiometric evaluations were audited separately. 268 samples (1.41%) were excluded due to missing data, primarily resulting from prolonged waiting times or participants being unable to complete the full test

**Conclusion:**

Alcohol consumption and self‐reported hearing impairment were linked to improvements in hearing and cognition, while age and smoking were risk factors. Data management implementation highlighted the necessity of multi‐stage quality control measures

